# The genetic pleiotropy of musculoskeletal aging

**DOI:** 10.3389/fphys.2012.00303

**Published:** 2012-08-08

**Authors:** David Karasik, Miri Cohen-Zinder

**Affiliations:** Faculty of Medicine in the Galilee, Bar-Ilan UniversitySafed, Israel

**Keywords:** aging, sarcopenia, tendon, bone, cartilage, musculoskeleton, pleiotropic genes, genome-wide studies

## Abstract

Musculoskeletal aging is detrimental to multiple bodily functions and starts early, probably in the fourth decade of an individual's life. Sarcopenia is a health problem that is expected to only increase as a greater portion of the population lives longer; prevalence of the related musculoskeletal diseases is similarly expected to increase. Unraveling the biological and biomechanical associations and molecular mechanisms underlying these diseases represents a formidable challenge. There are two major problems making disentangling the biological complexity of musculoskeletal aging difficult: (a) it is a systemic, rather than “compartmental,” problem, which should be approached accordingly, and (b) the aging *per se* is neither well defined nor reliably measurable. A unique challenge of studying any age-related condition is a need of distinguishing between the “norm” and “pathology,” which are interwoven throughout the aging organism. We argue that detecting genes with pleiotropic functions in musculoskeletal aging is needed to provide insights into the potential biological mechanisms underlying inter-individual differences insusceptibility to the musculoskeletal diseases. However, exploring pleiotropic relationships among the system's components is challenging both methodologically and conceptually. We aimed to focus on genetic aspects of the cross-talk between muscle and its “neighboring” tissues and organs (tendon, bone, and cartilage), and to explore the role of genetics to find the new molecular links between skeletal muscle and other parts of the “musculoskeleton.” Identification of significant genetic variants underlying the musculoskeletal system's aging is now possible more than ever due to the currently available advanced genomic technologies. In summary, a “holistic” genetic approach is needed to study the systems's normal functioning and the disease predisposition in order to improve musculoskeletal health.

Aging of the musculoskeletal system starts early and is detrimental to multiple functions of the whole organism, since it leads to disability and degenerative diseases. The age-related musculoskeletal changes are important in medical risk assessment and care because they influence the responses to treatment and outcomes of therapy. Challenges posed by this growing problem of health care and social medicine are well recognized and are a focus of efforts by many health professionals, as well as biomedical scientists.

There are two major problems that one faces while trying to disentangle the biological complexity of the musculoskeletal aging: (a) it is a systemic, rather than “compartmental,” problem, which should be dealt with accordingly, (b) the aging *per se* is neither well defined nor reliably measurable. A unique challenge of studying any age-related process is a need of distinguishing between the “norm” and “pathology,” which are interwoven in the aging. When another dimension is added, namely genetics underlying the system's functioning, even less is known about this aspect, and attempts to decipher genetic relationships between the system's components are few. By definition, genetic study of a complex system should explore pleiotropic relationships among the system's parts; however, this is challenging both methodologically and conceptually.

To disentangle the aging-related pathology from the homeostasis particular for aging steady-state, is a challenging task. Despite the multiple definitions of the aging process were proposed, there is no single agreed upon and reliable measurement (Karasik, 2011), therefore underlying molecular mechanism of aging is still not fully understood (de Magalhaes et al., 2009). The definition of aging is complicated by the occurrence of various diseases that modify body functions and tissue structures; these disease-related changes that are common in older persons are often hard to delineate from the aging process *per se* (Nair, 2005). Disease processes and environmental factors also need to be taken into consideration since they affect the rate of aging. Therefore, for our purpose, the “aging” will be considered as a changing environment in which the musculoskeletal system's homeostasis takes place. Aging-related factors provide an additional challenge for the study of genetic effects, since parameters such as co-expression of genes and penetrance of genetic variants can be “masked” by aging. It is necessary to detect genes with pleiotropic functions which are involved in the aging process, since this can provide insights into the potential biological mechanisms underlying inter-individual differences in susceptibility to the aging (Karasik, 2011). In turn, the disentangling effects of these genes in some of the facets of the musculoskeletal system might contribute to the knowledge related to the aging process of the entire organism.

Identification of genetic variants associated with traits related to the musculoskeletal system's physiology is now possible, due to the rapid development of various novel sequencing technologies, each aspiring to reduce costs to the point at which the genomes of individual humans could be sequenced as part of routine health care (Shendure et al., 2004), as well as the possibility to replicate associations in large human cohorts. Although many recent reviews have been dedicated to aging changes in the musculoskeletal system, most focused on the contribution of advanced age to one of the components of the system and/or on development of one of the musculoskeletal diseases—e.g., osteoporosis (Khosla et al., 2011), osteoarthritis (Loeser, 2010), sarcopenia (Rolland et al., 2008; Fielding et al., 2011), tendinitis (Rechardt et al., 2010) and similar. In turn, this perspective will focus mostly on the muscular part; although we do not attempt to assign the muscle a central role in the “musculoskeletal” system, it seems that the biological importance of the muscle is frequently overlooked while studying other components, and undeservedly so.

## Parts of the musculoskeleton in adulthood and aging

The term “musculoskeletal system” is most frequently used to describe the biomechanical relationships between muscle, tendon, fascia, bone, and cartilage, which are most evident in locomotion or mastication. However, these organs are interconnected at many other levels, including biochemically and micro-structurally. Musculoskeleton includes muscle, tendon, fascia, bone, and cartilage. Skeletal (striated) muscle is a tissue on its own, while other components of the musculoskeleton belong to the connective tissue and have different embryonic origin. Tendon, ligament, bone, and cartilage are connective tissues of mesenchymal origin. Morpho-functional components of the system also include fascia and aponeuroses, which are beyond the scope of the present review. It is important to note that many components of the skeletal muscle are also built by connective tissue. Muscle extracellular matrix (ECM) is often subdivided into epimysium (around the whole muscle), perimysium (around groups of muscle cells), and endomysium (around the muscle cell), and the basement membrane which is considered to be distinct from the endomysium. However, this simplified presentation does not fully explain the transmission of force from the myofiber to the tendon. Detailed studies of the transition zones between endomysium, perimysium, epimysium, and tendon are lacking.(Gillies and Lieber, 2011).

Muscle fibers are multinucleated, post-mitotic, highly differentiated cell agglomerates. Muscles of different functions are probably physiologically distinct; some are chronically active, perform antigravity functions, operate at long lengths, or are involved in rapid movements (Gillies and Lieber, 2011). Based on contractile and metabolic properties, muscle fibers are commonly classified as slow-twitch and fast-twitch, which are further subdivided to more aerobic (Type I) and the more anaerobic glycolytic (Type II), fibers. Adult fast/type-II fibers express one or more of the six type II sarcomeric myosin heavy chain (MHC) genes, while slow/type I fibers express β-MHC isoform (Resnicow et al., 2010). There are different types of collagen expressed during skeletal muscle development, although fibrillar types I and III predominate in adult endo-, peri-, and epimysium (Gillies and Lieber, 2011).

Under normal conditions, skeletal muscle is relatively quiescent, however, it harbors considerable regenerative capacity due to the presence of tissue-resident muscle stem cells known as the satellite cells (Sacco et al., 2010). However, with aging, cellular changes occur that reflect a reduced regenerative capacity of muscle (Collier et al., 2011). Common conditions of muscle aging include sarcopenia (muscle wasting), myosteatosis (gradual infiltration of muscle with fatty tissue), and fibrosis (accumulation of excess ECM). Morphologically, with advanced age, there are alterations in muscle morphology: the muscle tissue is progressively replaced by adipose and fibrotic tissue. Muscle mass is well maintained through the fourth decade of life (Nair, 2005) and shows a moderate decline between 50 and 60 years of age, followed by a more accelerated rate of loss beyond 60 years of age (Deschenes, 2004). Sarcopenia is manifested by decreases in muscle strength and muscle mass with age. The age-specific response to damage seen in muscles of older people includes higher protein expression for nuclear factor kappa B (Nf-κ B) and heat shock protein 70 (Mann et al., 2011). Senescence is associated with a decrement in the number of muscle fibers along with a reduction in the size of type II, but not type I fibers (Deschenes, 2004); the relative percentages of different fiber types appear to be unaffected by age (Deschenes, 2004).

Sarcopenia seems to occur by mechanisms that partly are unique to it and partly are common to other forms of atrophy (Mann et al., 2011). There are several potential causes of sarcopenia, among which the most prominent are age-related changes in the hormonal status, low levels of physical activity, reduced protein intake, and increased oxidative stress (Poehlman et al., 1995). Physiologically, after acute injury, such as after sports injuries, damaged or dead fibers are first removed by inflammatory cells, and they are then repaired or replaced by satellite cells (Cosgrove et al., 2009; Mann et al., 2011), which play a major role in post-natal muscle growth and repair. Successful regeneration of healthy muscle thus requires an inflammatory response with the migration and proliferation of fibroblasts, in order to produce new temporary ECM components, such as collagen types I and III, fibronectin, and others. The primary mononuclear cell in normal muscle ECM is the fibroblast, which is responsible for producing the majority of ECM components, including collagen, fibronectin, and proteoglycan (Gillies and Lieber, 2011). Fibroblasts are able to convert mechanical signals into altered gene expression [see review by Gillies and Lieber (2011)]. When tissue is damaged, fibroblasts migrate into the damaged area and begin to produce and remodel the ECM in response to pro-fibrotic cytokines such as transforming growth factor-β (Mann et al., 2011). They serve to stabilize the tissue and act as a scaffold to which the new fibers can migrate, which is necessary for effective repair of damaged tissues.

However, with advanced aging, the chronic inflammatory response drives unrestrained wound healing and tissue fibrosis, characterized by excessive and persistent fibrin/fibrinogen deposition (Mann et al., 2011). Alternatively-activated macrophages have been shown to promote fibrosis in several different pathogenic conditions (Wynn, 2008). Satellite cells can also differentiate into fibroblasts (Brack et al., 2007).

Fibrin(ogen) can also directly stimulate the expression of collagen; in turn, collagen type I can markedly suppress differentiation of C2C12 cells (the cell line established to become muscle), thus muscle fibrosis is seen as a self-perpetuating mechanism of collagen overproduction (Mann et al., 2011). One might hypothesize that, similar to sarcopenia, advanced muscle fibrosis will have a detrimental effect also on bones, both due to diminished muscle strain and a lack of the myokines due to insufficient population of the myocytes.

With “normal” aging, there is a marked increase in the formation of advanced glycation end-products (AGEs). AGEs are produced by the spontaneous non-enzymatic glycation of proteins (Grillo and Colombatto, 2008) and crosslinking. These collagen modifications increase the stiffness of muscle connective tissue, thereby contributing to impaired muscle function in the older person (Mann et al., 2011). AGEs do not only affect muscles: the accumulation of AGEs in articular cartilage with aging (independent of diabetes) may be attributed in large part to the very slow turnover of cartilage matrix components (Lotz and Loeser, 2012). It has been suggested that in bones, AGEs enhance osteoclast-induced bone resorption, and AGE modification of bone proteins disturbs bone remodeling (Hein et al., 2006). Interestingly, it was shown that AGE-specific binding sites are present in cultured osteoblast-like cells, thus AGEs can regulate proliferation and differentiation of osteoblastic lineage (Hein et al., 2006).

In an alternative path to muscular degeneration with age, intermuscular adipose tissue content and intramyocellular lipid deposits increase, and adipose tissue appears to replace muscle tissue (Song et al., 2004). Muscle lipid content can be reliably measured as muscle attenuation with computed tomography (CT) (Miljkovic et al., 2009). Intermuscular adipose tissue may accumulate in skeletal muscle of elderly people as a result of satellite cells differentiating into adipogenic cells. In aged muscle, there is also an accumulation of the intramuscular, extracellular fat: these fatty deposits are generally localized to the perimysium (Gillies and Lieber, 2011) (more details on the intermuscular adipose tissue are provided below).

### Tendons

Tendons are dense, fibrous connective tissue bands that connect muscle to bone, transmitting the muscular forces to perform movement. Fibroblasts aid in tendon development and maintenance (Gillies and Lieber, 2011). Tendons are built mostly of collagen; however, expression of fibrillar collagens varies along the bone insertion: tendon fibroblasts expressed type I collagen, fibrochondrocytes in the transitional tissue between tendon and bone expressed type II and X collagen, and osteoblasts in bone again expressed type I collagen (Thomopoulos, 2011). The proteoglycans found in tendons, including decorin, fibromodulin, biglycan, and lumican, act to lubricate and organize collagen fiber bundles (Ito et al., 2010). Tendon and perimysium both contain primarily type I collagen, and the primary proteoglycan for both structures is decorin. Perimysium seems to be continuous with tendon (Gillies and Lieber, 2011): collagen in tendon becomes much more organized than collagen in perimysium; high tensile loads on the tendon may organize collagen fibers to align with the muscle axis (Gillies and Lieber, 2011). With aging, enzymatic and nonenzymatic cross-linking is observed. Although cross-linking might signify an aberrant collagen, and thus mechanically less stable tendon, some proposed this might be a mechanism to maintain the mechanical properties of the tendon into advanced age (Couppe et al., 2009). Generally, tendon damage from overuse or degeneration is a common clinical problem in geriatrics and orthopedics, because damaged tendon heals very slowly and rarely recovers completely (Ito et al., 2010).

### Bone

Bones develop during embryonic period by either intramembranous ossification (flat bones) or endochondral ossification. The majority of the bones develop endochondrally; those include the long, short, and irregular bones, thus most of the bones are developmentally related to cartilage. Bone cells include osteoclasts, osteoblasts, and osteocytes, whose simplified roles can be described as bone resorption, bone formation, and sensoring/mechanosensitivity, respectively. Bones are built by two compartments, cortical and cancellous (trabecular). Cortical bone consists of a repeating unit, named osteon; the unit structure of cancellous bone is a trabeculum (with a built-in “hemi-osteon”). Muscle and tendon interconnect to transmit force to bone, and the tendon inserts to the bone frequently via fibrocartilage (enthesis) (Gillies and Lieber, 2011; Thomopoulos, 2011). The fibrocartilaginous enthesis forms partly through endochondral ossification (Thomopoulos, 2011). The decrease in collagen fiber orientation in enthesis along with a functionally graded insertion of tendon to bone are major determinants of ensuing stiffness. The linear increase in mineral accumulation on collagen fibers provides significant stiffening of the fibers, but only for concentrations of mineral above a “percolation threshold” corresponding to formation of a mechanically continuous mineral network (Thomopoulos, 2011).

Osteoporosis is among the most common skeletal disorders; it is characterized by low bone mineral density (BMD) and an increased risk of fragility fractures. BMD predictably declines with advanced age; it is the best clinical predictor of future osteoporotic fracture risk. However, BMD measures are relatively ineffective in predicting the skeleton's ability to withstand the forces that produce fractures (Christiansen and Bouxsein, 2010). BMD is also a complex trait controlled by multiple environmental and genetic determinants with individually modest effects, which makes it a difficult phenotype to use in biological exploration. Degenerative bone diseases have been described in detail in a number of recent reviews (Michaelsson et al., 2007; Shanmugarajan et al., 2007) and are therefore not discussed further here.

### Joints

Joints can be classified into three groups based upon how the bones within the joint are connected: fibrous, cartilaginous, and synovial (Benjamin et al., 1995). Joint tissues include articular cartilage, ligaments, and menisci, which are frequently surrounded by synovial membrane and fluid. Articular cartilage contains only post-mitotic cells of mesenchymal lineage; with a very low rate of replication during adulthood, which makes them vulnerable to trauma and most affected by the aging process (Lotz and Loeser, 2012). The major collagens of the cartilage are Type II (characteristic of chondrocytes) and Type X (characteristic of hypertrophic chondrocytes) (Thomopoulos, 2011). Cells of cartilage, chondrocytes, and osteoblasts are well interrelated: notably, the chondrocytes frequently transdifferentiate into osteoblasts. Cartilage thus has an innate ability to ossify. Fibrous suture joints in the skull are eventually replaced by bone in childhood. Cartilaginous joints are found in growth regions of the long bones (Wu et al., 2011), where they are also gradually replaced by bone during childhood. A highly prevalent change in aging cartilage is cartilage calcification—by deposition of calcium-containing crystals, mainly calcium pyrophosphate and basic calcium phosphate due to increased pyrophosphate production by chondrocytes from aged cartilage (Lotz and Loeser, 2012).

Notably, similar to cartilage, there is an increased incidence of *heterotopic ossification*, a condition where bone forms within muscles, with aging; it can cause muscle necrosis. The new bone seems to actually form in connective tissue between muscle planes and not in the muscle itself. Heterotopic bone formation occurs also in ankylosing spondylitis, hypertrophic osteoarthrosis, and diffuse idiopathic skeletal hyperostosis. A useful review on inherited human diseases of heterotopic bone formation was recently published (Shore and Kaplan, 2010). Degeneration of other joint tissues, such as ligaments and menisci has been examined at some level (Pauli et al., 2011), however at a lesser extent than the aging of cartilage.

## Etiology of common musculoskeletal age-related conditions

The musculoskeletal system can be regulated by multiple biological mechanisms, including anatomical (anabolic), physiological (anaerobic threshold, hormones), or even behavioral (desire to exercise, pain tolerance) pathways. To get a better understanding of the system's molecular organization and higher-level function, novel pathways with pleiotropic effects of the musculoskeletal system's components should be discovered. Pleiotropy is usually implied when a single gene seems to control multiple phenotypic traits. Strictly speaking, this would be a “direct” pleiotropy; usually, there might be a mediation of a gene's effect via one trait onto the other, or even a confounding by some third factor.

Each compartment of the musculoskeletal system—muscle, tendon, bone, and cartilage—undergoes aging. Despite years of intense studies, we are still relatively ignorant of the molecular basis of the majority of genetically-influenced musculoskeletal phenotypes. In terms of genetics, each degenerative condition is a common and complex disease; even some “rare” syndromes, such as Duchenne Muscular Dystrophy (DMD) or rheumatoid arthritis (RA), are also complexly-regulated traits. It can be anticipated that with the broad use of next generation sequencing (NGS), most causative genes for rare musculoskeletal diseases will be identified in the next few years (Laing, 2012). “Rare” diseases are thus expected to provide some insight into the genetics of common diseases of the musculoskeletal aging, since they share etiology with the latter [see, for example, *LRP5* gene, originally discovered in the rare diseases such as high-bone-mass trait and osteoporosis-pseudoglioma (Boyden et al., 2002; Little et al., 2002) but further being associated with BMD and fracture risk in the general population (Ferrari et al., 2005; Kiel et al., 2007; Estrada et al., 2012)].

Degenerative musculoskeletal process (Lotz and Loeser, 2012) seems to be a reflection of a general aging, which can be seen as disorganization of the cellular homeostasis mechanisms. Survival and normal function of post-mitotic cells like muscle fibers and mature articular chondrocytes depends on their ability to cope with persistent wear and tear. Conceptually, age-related pathologies originate from limitations in the maintenance and repair mechanisms of DNA, by e.g., anomalies in the antioxidant mechanisms that contribute to the detoxification of reactive oxygen species (ROS) (Lotz and Loeser, 2012).

### Telomeres and mitochondria in musculoskeletal aging

Shortening of the telomeres and mitochondrial somatic mutations were suggested to have an impact on aging skeletal muscle [see recent studies by Dillon et al. (2012) and Payne et al. (2011)]. The shortening of the telomeres at the ends of chromosomes is considered to be a measure of cell senescence; it has been associated with age-related disease and mortality (Wheeler and Kim, 2011). It was also shown that centenarians and their offspring maintain longer telomeres compared with controls and that longer telomeres are associated with protection from age-related diseases, better cognitive function, and lipid profiles of healthy aging (Atzmon et al., 2010; Wheeler and Kim, 2011). A recent study identified a common haplotype of *hTERT*, coding for the human telomerase reverse transcriptase gene, that is enriched in centenarians and associated with longer telomere length (Atzmon et al., 2010). Aging of mitotically active human tissues with high turnover, including blood, liver, skin, testis, and kidneys, is accompanied by telomere shortening (Aikata et al., 2000). In contrast, analysis of telomeres in skeletal muscle during aging reveals only a mild shortening (Decary et al., 1997; Renault et al., 2002), presumably reflecting the low rate of proliferation of myogenic progenitors and muscle tissue turnover during normal aging (Sacco et al., 2010). Telomere length was shorter in women with osteoporosis. In bones, telomere shortening and telomerase activity have been linked to *in vitro* osteoblast senescence and to increased secretion of pro-inflammatory cytokines (Valdes et al., 2007). Telomere shortening can also be promoted by extrinsic or “stress-induced” factors such as the chronic effects of oxidative damage and inflammation (Loeser, 2010; Lotz and Loeser, 2012).

Mitochondrial dysfunction and excessive ROS production contribute to oxidative stress and chronic inflammation of the musculoskeleton. For example, ROS-mediated adverse effects of diabetes are seen on bone formation and maintenance, as ROS greatly influence the generation and survival of osteoclasts, osteoblasts, and osteocytes (Manolagas, 2010). Excessive levels of ROS can contribute to aging in many tissues (Ahima, 2009): in muscles, ROS may trigger different signaling pathways leading to diverging responses, including either autophagy or apoptosis in the tissues (Barbieri and Sestili, 2012). In joints, there is increased apoptosis in OA chondrocytes following exposure to oxygen radicals; also, oxidized low-density lipoprotein (LDL) could form when LDL present in synovial fluid reacts with ROS (Lotz and Loeser, 2012).

### Molecular cascades involved in the inflammation

A direct relationship exists between aging and increasing incidence of chronic diseases, which is in part due to the underlying chronic inflammatory state. However, the molecular mechanisms underlying this chronic inflammatory condition, such as local infiltration by inflammatory cells, are presently unclear, as well as whether aging-related inflammation can be triggered by cellular senescence.

The circulating concentrations of cytokines and C-reactive protein are often elevated in people with age-related diseases, including obesity and Type 2 diabetes, osteoporosis, RA, and Alzheimer disease (Tan et al., 2007; Peake et al., 2010). Some form of inflammatory response is necessary to repair damaged tissues effectively; however, chronic inflammatory responses drive unrestrained wound healing and fibrosis especially in aging (Mann et al., 2011). Intermuscular adipose tissue may accumulate in skeletal muscle of elderly people as a result of satellite cells differentiating into adipogenic cells. Satellite cells can also differentiate into fibroblasts in “old” muscles (Le Grand and Rudnicki, 2007). Both adipocytes and fibroblasts, which replace myoblasts, produce proinflammatory cytokines, which in turn may contribute to the inflammatory state of skeletal muscle in the elderly.

Local inflammation in adipose and synovial tissues is a causative factor in some of the degenerative changes in these and neighboring tissues. Anti-inflammatory cytokines (e.g., IL-4, IL-10, IL-13) play central role in resolving local inflammation and repairing muscle tissue following injury (Peake et al., 2010). It is unclear whether “overflow” of inflammatory cytokines and reactive oxygen/nitrogen species from these tissues and their “spill-over” into the circulation can cause inflammation in neighboring tissues, such as bone. Systemic inflammation in elderly people has been hypothesized for a long time to produce a background milieu (Finch and Ruvkun, 2001; Peake et al., 2010), which affects even distant organs.

Paradoxical relationships between exercise, which triggers the skeletal muscle inflammation, and a positive effect of the exercise on the bones, are of a particular interest. For example, inflammatory cytokines IL-6 and IL-7 are released as a result of physical activity and ensuing muscle contraction; they are, however, activating bone resorption and turnover. On the contrary, an over-secretion of interleukin-15 occurs due to resistance exercise, and its beneficial effects involve a decrease in adiposity and increase in bone mass (Quinn et al., 2009).

### Role of muscles in energy metabolism and insulin sensitivity

The adipose tissue is an endocrine organ producing a variety of factors which regulate energy metabolism and insulin sensitivity (Sell et al., 2006). There are distict types of the adipose: white, brown; subcutaneous, visceral, and inter/intramuscular tissue. Adipose tissue protects other cell types from “lipotoxicity” by providing a safe haven for surplus energy. On the other hand, increased adipose tissue mass is associated with insulin resistance, systemic dyslipidemia, hyperglycemia, hypertension, and components of the metabolic syndrome (Dandona et al., 2005; Despres, 2006). Lately, Schafer et al. (2010) found that fat infiltration of muscle was higher in adults with diabetes or impaired glucose metabolism than in those with normal glucose levels (*P* < 0.001). Furthermore, fat infiltration of muscle was independently associated with a 19% increased risk of incident clinical fracture (hazard ratio = 1.19; 95% confidence interval = 1.04–1.36); this association did not differ across glucose metabolism groups. Skeletal muscle is a major depot responsible for glycogen synthesis; therefore, there is an ongoing interest in the metabolic interplay between adipocytes and skeletal myocytes. There are suggestions of a negative crosstalk between body fat and skeletal muscle, which results in disturbances of insulin signaling in skeletal muscle as well as insulin resistance (Sell et al., 2006). Direct adipocyte–myocyte interaction was first explored by Dietze et al. (2002), who demonstrated a direct cross-talk between human adipocytes and myocytes in a co-culture model in which both cell types shared the same medium. Co-culture with adipocytes resulted in insulin resistance in skeletal muscle cells (Dietze et al., 2002; Dietze-Schroeder et al., 2005). There is an effect of local fat also on bone: using normal human osteoblasts co-cultured with differentiating pre-adipocytes *in vitro*, Elbaz et al. showed that inhibition of fatty acid biosynthesis prevents adipocyte lipotoxicity on osteoblasts (Elbaz et al., 2010). After 3 weeks in co-culture, osteoblasts showed significantly lower levels of differentiation and function, with lower mineralization and expression of alkaline phosphatase, osterix, osteocalcin, and Runt-related transcription factor 2 (Runx2).

Evidence has been presented that adipose tissue communicates with the rest of the body not only via free fatty acids (FFAs) but also through adipose-derived cytokines (adipokines) (Sell et al., 2006), which have been implicated in the impairment of insulin sensitivity. On the contrary, adipokines leptin and adiponectin have each been shown to increase fatty acid oxidation and decrease in triglyceride storage in muscle, which may explain, in part, the insulin-sensitizing effect of these cytokines (Dyck, 2009). Recently, Tenta et al. (2012) explored the relationship of adiponectin with bone mass indices and bone metabolic markers in middle-aged post-menopausal women without diabetes, and showed significant associations with osteoprotegerin and IGF1 levels, suggesting an anabolic role of adiponectin. Together with the co-culture experiments, these observations may contribute in the understanding of the interplay between adipose tissue derived hormones and bone metabolism (Tenta et al., 2012).

Koonen et al. (2010) provided insight into the mechanisms by which aging becomes a risk factor for the development of insulin resistance in middle-aged mice, demonstrating that limiting skeletal muscle fatty acid transport is an effective approach for delaying the development of age-associated insulin resistance and metabolic disease during exposure to a high-fat diet. As opposed to the high-fat diet, caloric restriction is frequently used to improve function in aged animals. Thus, Selman et al. (2009) established that *S6K1*^−/−^ mice live longer and show improved health in later life than control mice. In these mice, the gene S6 Kinase 1 (*S6K1*) was inactivated. *S6K1* is a ribosomal S6 kinase, a component of the nutrient-responsive mammalian target of rapamycin (mTOR) signaling pathway. Microarray data analysis helped to characterize the gene-regulatory changes accompanying the long-lived animals compared with wild-type mice. For example, increased expression of genes associated with pathways known to be associated with aging and with caloric restriction (*Ppargc1a, Ppara, Foxo1, Foxo3a, Pdk4, Glut1, Sirt1*, and *Ucp3*), was observed in the muscle of *S6K1*^−/−^ mice. Down-regulation of ribosomal S6 protein kinase signaling was reported to regulate mammalian lifespan supposedly by increasing activity of AMP activated protein kinase (AMPK) (Selman et al., 2009), a master regulator of cellular energy homeostasis (Hardie, 2007). AMPK inhibits the mTOR pathway, an important regulator of growth control and metabolism. Skeletal muscle mitochondrial uncoupling activates AMPK (Gates et al., 2007; Neschen et al., 2008), and in turn, AMPK has been shown to regulate energy metabolism by modulating the activity of the histone/protein deacetylase SIRT1 (Canto et al., 2009), a molecule supposed to be involved in gene expression changes that mediate the increase in longevity induced by caloric restriction (Ruderman et al., 2010).

## Paracrine and endocrine crosstalk between muscle and its “neighbors”

There are several pathways with a plausible pleiotropic effect on musculoskeletal system's homeostasis, which seem to be among major players in the aging of musculoskeleton.

### Growth factors

The contribution of growth factors to the muscle and other parts of the system at different stages of development and aging, is well documented. Growth hormone (GH) is an important regulator of different physiological processes necessary for somatic growth and development, starting with the differentiation of muscle and bone cells, as well as metabolism of lipids and carbohydrates (Perrini et al., 2010). GH regulation and signaling can occur directly, through activation of specific GH receptors (Giustina et al., 2008), or indirectly, through insulin-like growth factor 1 (IGF-1) (Laviola et al., 2007). IGF-1 is produced primarily by the liver as an endocrine hormone in response to GH stimulation (Ohlsson et al., 2009) or in target tissues in a paracrine or autocrine fashion under the control of systemic hormones (Laviola et al., 2007; Kapoor et al., 2008). It was shown that the changes in GH and IGF-1 secretion that occur with aging are paralleled by a progressive loss of muscle mass and BMD (Bohannon, 1997). The contribution of IGF-1 to the maintenance of parts of the musculoskeleton is well documented. Mechanical stimulation of bone cells may induce elevated levels of IGF-1, which signals the differentiation of osteoblasts into osteocytes (Schmid et al., 1980); which maintain bone mass in response to normal load (Bonewald and Johnson, 2008). IGF-1 levels are positively correlated with muscle protein synthesis rates, specifically myofibrillar protein (actin and myosin filaments) and MHC synthesis (Waters et al., 2000). Overexpression of insulin-like growth factor and the simultaneous loss of myostatin in muscle *in vivo* have been found to have synergistic effects on myofiber growth and lessened fibrosis (Mann et al., 2011). The anabolic response to IGF-1, measured as proteoglycan or collagen synthesis, declines in human articular chondrocytes from older donors with increasing age (Lotz and Loeser, 2012).

Other growth factors regulating muscle mass include mechano-growth factor, myostatin, vascular endothelial growth factor (VEGF), or hepatocyte growth factor (HGF). In skeletal muscle, at least two different IGF-1 isoforms are expressed due to alternative splicing of the primary IGF-1 transcript (Goldspink, 2004). For the overview of growth factors and cytokines secreted by muscle, as well as their potential effects on bone metabolism, the reader is referred to a recent review by Hamrick (2011).

Mechano-growth factor (MGF) is a member of the IGF-1 superfamily that is induced in response to physical activity (Wu et al., 2011). It has a marked ability to induce satellite cell fusion for muscle repair and maintenance. Recent findings of up-regulation of MGF in prostatic cancer cells might highlighted its role also in cancer biology (Armakolas et al., 2010); this might suggest that caution should be exercised for its therapeutic use in older men (Thomis and Aerssens, 2012). Recently, Juffer et al. (2012) investigated whether mechanical loading by pulsating fluid flow modulates the messenger RNA (mRNA) and/or protein levels of muscle anabolic and metabolic factors in MLO-Y4 osteocytes. They showed that loaded MLO-Y4 osteocytes differentially express anabolic factors involved in the adaptive response of muscle to mechanical loading (i.e., IGF-1, MGF, VEGF, and HGF). Similarly to muscle fibers, mechanical loading enhanced expression levels of these growth factors in primary bone cells. The authors concluded that osteocytes respond to mechanical loading by producing more VEGF and HGF, which suggests that these proteins previously played unrealized roles in bone remodeling mediated by loading.

Fibroblast growth factor 2 (FGF2) is another polypeptide growth factor that stimulates satellite cells (a.k.a. muscle precursor cells, MPCs). MPCs isolated from sarcopenic animals exhibit a decreased proliferative response to FGF2 (Jump et al., 2009). FGF2 is also a well-known osteogenic factor; it has positive effects on bone formation in estrogen-deficient rodents (Hamrick, 2012).

### Transforming growth factor-β (TGF β) superfamily, myostatin, and activin receptors

TGFβ is a multifunctional protein that controls proliferation, differentiation, and other functions in many cell types. In the muscle, TGFβ seems to play an important role in aging-associated fibrosis and muscle impairment. Overexpression of active Notch2 in C2C12 cells (the cell line established to become muscle) prevents TGFβ from inducing the expression of collagen I, whereas transient knockdown of Notch2 by siRNA in cultured myoblasts results in the differentiation of C2C12 myoblasts into myofibroblastic cells that express fibrotic markers (Mann et al., 2011). For the recent review on role of TGFβ in osteoblast differentiation and bone formation, see Chen et al. (2012). The review also highlights the different modes of cross-talk between TGF-β/BMP and the signaling pathways of MAPK, Wnt, Hedgehog, Notch, and FGFs in osteoblast differentiation and bone formation (Chen et al., 2012). The role of TGFβ in muscle fibrosis of aging has been the subject of many studies [see review Dequeker et al. (2003)]. Activated TGFβ induces fibroblasts to produce type I collagen, fibronectin, and connective tissue growth factor (cTGF) and suppresses matrix metalloproteinases (MMPs). Notably, MMP levels in uninjured muscle are generally low, but secreted MMPs can degrade type IV collagen, fibronectin, proteoglycans, and laminin (Gillies and Lieber, 2011). When repeated muscle injury occurs, elevated TGFβ continues to stimulate ECM production and eventually leads to a fibrotic response (Gillies and Lieber, 2011). Logically, TGFβ inhibitors can be used to reduce aging-associated fibrosis in skeletal muscle (Mann et al., 2011).

A lot of research in the last decade was focused on myostatin (growth differentiation factor 8, GDF8), a secreted TGFβ protein family member that inhibits muscle differentiation and growth. Myostatin is produced primarily in skeletal muscle cells, circulates in the blood and acts on muscle tissue, by binding a cell-bound receptor called the activin type II receptor [for the recent review, see Hamrick (2012)]. Thus, elevated levels of myostatin expression are evident in disuse atrophy and cachexia (of cancer and AIDS) (Hamrick, 2011). Contrarily, inactivating mutations of the myostatin *(MSTN*, a.k.a. *GDF8*) gene induce a hypermuscular phenotype in mammals, with pronounced effect on bones (Hamrick et al., 2003). Importantly, myostatin is released from muscle during traumatic and catabolic conditions that may inhibit and suppress bone repair. Lee and McPherron (2001) previously showed that inhibition of myostatin by genetic elimination using knockout mice or by increasing the amount of the propeptide follistatin, resulted in greatly increased muscle mass. Follistatin (also known as activin-binding protein) is an autocrine glycoprotein that is expressed in nearly all tissues of higher animals (Tortoriello et al., 2001). Follistatin is known to regulate myostatin activity and muscle growth (Lee and McPherron, 2001). Kota et al. (2009) showed that regulation of follistatin via gene therapy also resulted in muscle growth and increases in strength in non-human primates. The authors agreed with the findings in mice detected by Lee and McPherron (2001), so the findings of both studies point out that gene therapy may improve muscle mass and function in patients with certain degenerative muscle disorders.

Myostatin not only regulates the growth of muscle cells, but also fibroblast activation and hence the progression of fibrosis in the muscle. In the absence of myostatin, the improved regeneration and decreased fibrosis took place (Mann et al., 2011). It appears that myostatin also regulates the structure and function of tendon tissues, as the stiffness of tendons is 14 times higher in myostatin-deficient mice than in the wild-type controls (Mendias et al., 2008). Importantly, myostatin expression is increased by glucocorticoids; this suggests that myostatin is required for the catabolic effects of glucocorticoids, leading to muscle atrophy (Gilson et al., 2007). It is well known that glucocorticoid-induced enhanced proteolysis also corresponds to increase in bone loss (Kanis et al., 2007).

Myostatin binds to the soluble activin type IIB receptor (ACVR2B) and to a lesser extent to activin type IIA receptor (ACVR2A), which act as myostatin antogonists and thus exert differential anabolic activity in bone and muscle (Lee et al., 2005). Digirolamo et al. presented data on muscles and bones in three groups of mice given either one of two types of ACVR2 or placebo (Digirolamo et al., 2011). Their study findings suggest that both ACVR2 and ACVR2B produce anabolic effects on muscle and bone; however, ACVR2's effect was greater on bone than muscle, whereas ACVR2B's effect was greater on muscle than bone. The activin receptor types and their related function in the musculoskeletal system are summarized in Table 1. *In vitro* and *in vivo* studies have demonstrated that both activin A and its antagonist follistatin play opposite roles in bone formation (Eijken et al., 2007): follistatin increases bone formation in mice, by inhibiting activin A *in vivo* using a decoy soluble activin A receptor (Vallet et al., 2010). Activin A treatment can inhibit mineralization in cultured osteoblasts. Loss-of-function of activin type I receptor (ACVR1) in osteoblasts increases bone mass and activates canonical Wnt signaling through suppression of Wnt inhibitors sclerostin (SOST) and DKK1 (Kamiya et al., 2011). A point mutation in ACVR1 can cause fibrodysplasia ossificans progressiva (FOP) in which ectopic ossification occurs in skeletal muscles and deep connective tissues (Lucotte et al., 2009; van Dinther et al., 2010).

**Table 1:** **Activin receptor types and their function in the musculoskeletal system**.

**Activin receptors/sub-units**	**Function in bone/muscle**	**References**
**ACTIVIN TYPE I RECEPTOR**		
ACVRA1	Essential for skeletal development.	Chen et al., 2004; Lucotte et al., 2009; van Dinther et al., 2010
	Involved in ossification of muscles and joints in fibrodysplasia ossificans progressiva (FOP) disease, through mutations in ACVR1 and noggin gene.	
	A mutation causes endothelial cells to transform to mesenchymal stem cells and then to bone	
ACVR1B	Involved in muscle strengthening	Windelinckx et al., 2011
ACVR1C	Is used as an antagonist of myostatin, which inhibits muscle cells proliferation	Digirolamo et al., 2011
**ACTIVIN TYPE II RECEPTOR**		
AVCR2A	Regulates muscle growth and bone formation	Lee et al., 2005; Lotinun et al., 2010
AVCR2B	Involved in the signaling pathway essential for initiating osteoblast differentiation	Liu et al., 2012

### Vitamins

The connection between muscle and bone can be regulated by vitamins, most prominently, by vitamin D. Vitamin D, a key regulator of bone metabolism, is also known to be significantly associated with muscle strength: a lack of vitamin D, which is associated with aging, can cause myopathy. In the elderly, vitamin D deficiency is linked to muscle weakness, increased body sway, and increased susceptibility to falls and fractures. The mechanisms underlying the effect of vitamin D on muscle strength are not fully understood; thus, vitamin D action requires activation of the vitamin D receptor, which is widely distributed in various tissues including skeletal muscle (Endo et al., 2003; Grundberg et al., 2004; Windelinckx et al., 2007). Gilsanz et al. demonstrated that serum 25OHD was inversely related to the percent fat of skeletal muscle, a relation that was independent of body mass or subcutaneous and visceral fat depots. Compared with women with normal serum 25OHD concentration, vitamin D-insufficient women had approximately 24% greater muscle fat infiltration (but no significant differences in the cross-sectional area of their thigh muscles) (Gilsanz et al., 2010). Non-straightforward seems to be the relationship between the serum levels of vitamin D and osteoarthritis. Vitamin D has been shown to stimulate synthesis of proteoglycan by mature articular cartilage *in vitro*, and this suggests that vitamin D may directly affect articular cartilage metabolism, but its relationships with the clinical OA seem to be confounded by other aging-related processes.

Other vitamins are also have roles in the musculoskeleton regulation, however, less is known. Vitamin K deficiency has been linked to a variety of age-associated conditions, including loss of BMD or increased fracture risk, arterial calcification, and inflammation (Shea et al., 2009). Bone-related conditions and arterial calcification have been most widely studied in relation to vitamin K availability (McCann and Ames, 2009), while there are virtually no data on the vitamin K effect on skeletal muscle. On the contrary, a role of Vitamin E in the muscle physiology seems to be well known. Vitamin E could reduce muscle inflammation (Tiidus and Houston, 1995). Since a large majority of vitamin E is found in adipose tissue, this might reflect anti-oxidant properties of vitamin E and indicate its supplementation can help reduce muscle damage caused by free radicals. Interestingly, mice with a genetically-induced vitamin E deficiency, have high bone mass as a result of a decrease in bone resorption. Vitamin E thus decreases bone mass by stimulating osteoclast fusion according to Fujita et al. (2012).

### Menopause and sarcopenia: is there evidence for a causal relationship?

Menopause is associated with a decline in estrogen levels, which is translated into an increase in fat mass, as well as a decrease in bone density, muscle mass, and muscle strength. Decline in muscle mass (sarcopenia) is frequently observed in post-menopausal women (Messier et al., 2011). During the menopausal transition, there is a sharp fall in estrogen levels, therefore it has been suggested that changes in female estrogen levels may play a role in the development of sarcopenia during menopause. Estradiol (E2), together with estriol (E3) and estrone (E1), belong to estrogens. So far, there is limited evidence whether the loss of estradiol negatively affects muscle mass and physical function. Ratiani et al. (2012) were exploring lately the influence of estrogen on the intensity of oxidative metabolism in women of reproductive and menopausal age. For this study, two groups of women—less than 45 years old (reproductive age) and older than 45 (menopausal age) were tested. It was found that the physiological reduction in estrogen levels during menopause by itself contributes to impairment of oxidative metabolism and intensification of inflammation, oxidative stress, hypoxia, and related conditions.

Aging is associated with a loss of sex hormone not only in women but also in men; this phenomenon is called the “andropause” or the “male menopause.” In men, reduction in the levels of the androgen testosterone can trigger declines in muscle mass, bone mass, and overall physical function (Horstman et al., 2012). It was previously shown that administration of supplemental testosterone orally to older relatively hypogonadal men resulted in an increase in muscle mass and a decrease in body fat (Wittert et al., 2003). Lately, the Toledo Study for Healthy Aging (Carcaillon et al., 2012) found that there are sex differences in the association between serum levels of free testosterone and frailty (a syndrome of aging-related loss and dysfunction of skeletal muscle and bone) in an elderly population. The authors suggested that although the age-associated decline in testosterone occurs in both men and women, this decline does not arise to the same extent in both sexes, suggesting a possible differential impact on frailty according to sex.

**Figure 1:**
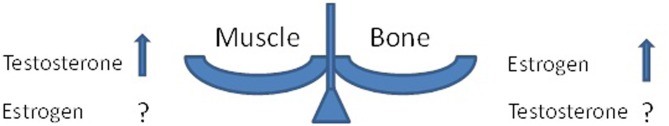
**Possible changes in muscle and bone in response to sex hormone levels**.

Adverse effects of menopause on bones are well known (Kanis, 1996); less obvious are effects of “andropause” on bones (Ferrari et al., 2005). The uncertainties of the roles of major sex hormones in muscle and in bones are illustrated in Figure 1. Recently, a Finnish study investigated a link between the estrogen replacement therapy (ERT) and skeletal muscle transcriptome (Ronkainen et al., 2010). Long-term use of ERT was associated with subtle differences in muscle transcript profiles and a better muscle fiber composition, although no differences were observed in mitochondrial DNA copy number or oxidative capacity per muscle cross section. Similar to the menopausal etiology of osteoporosis, the role of estrogen has been a long-standing theme in osteoarthritis research, since OA is more common in women than in men (a ratio of ~3:1). OA often begins around the time of menopause, and incidence rises faster in menopausal women than in men of the same age. Joint pain is a common complaint at the time of menopause or ERT withdrawal.

In addition to changes in androgen and estrogen levels, another important player in the development of sarcopenia in elderly population might be myostatin, a secreted TGFβ family member that inhibits muscle differentiation and growth (as detailed above). The relationship between testosterone and myostatin in the humans is not fully understood, although testosterone is known to reduce myostatin release in the muscle (Kovacheva et al., 2010). Studies indicated that myostatin release in the muscle is not necessary related to sarcopenia. Ratkevicius et al. (2011) showed that serum concentrations of myostatin and myostatin-interacting proteins do not differ between young and sarcopenic elderly men. The authors suggested that altered serum concentrations of myostatin-interacting proteins might not contribute to sarcopenia with the possible exception of follistatin-related gene (FLRG). FLRG encodes a secreted glycoprotein that is highly homologous to follistatin and binds activins and bone morphogenetic proteins (Wang et al., 2003). In a different study, in young and old men treated with graded doses of testosterone, myostatin levels were significantly higher on day 56 than on baseline in both young and older men. Notably, changes in myostatin levels were significantly correlated with changes in total and free testosterone only in young men, probably suggesting that response to testosterone is affected by age (Lakshman et al., 2009).

## Genetics of sarcopenia and other common musculoskeletal age-related conditions: evidence of pleiotropy?

### Relationship between muscle and bone mass in adults

Skeletal muscle has a close functional relationship with bone, starting in embryonic period. Developmentally, osteoblasts and muscle cells derive from a common mesenchymal precursor, the pluripotent mesenchymal stem cells. For example, the C2C12 cell line is established to become muscle but can be induced to differentiate into osteoblasts in the presence of BMP-2 (Darcy et al., 2012).

Studies of both humans (adults and children) and laboratory animals have documented a strong, positive correlation between muscle strength and bone mass (Gilsanz et al., 2006). Developmentally, this process is a reflection of allometry (usually defined as the covariance between the form, shape, and size of body part and size of the whole organism) (Karasik and Kiel, 2010). Further in the life, muscle atrophy is concomitant with the observed bone loss (Judex et al., 2004). There is a “biomechanical” explanation for this phenomenon. Mechanical loads activate new bone formation on cortical and trabecular surfaces; strain can activate some bone cells, which then respond with gene activation, increased metabolism, growth factor production, and building the matrix (Forwood, 2001; Frost, 2003). For example, in their recent study, Zhang et al. (2012) found that osteogenic response of bone mesenchymal stem cells to continuous mechanical strain is dependent on the signaling of extracellular regulated protein kinase (ERK) 1/2 and Runx2. Runx2 is a key transcription factor known to regulate the differentiation and/or function of osteoblasts. It is also an important mediator of the ability of metastatic breast cancer cells to directly modulate both osteoclast and osteoblast function. Sears et al. (2007) showed there is an evolutionary association between Runx2 repeats (changes in glutamine-alanine tandem-repeat ratio) and facial skull length in carnivores suggesting Runx2 tandem repeats providing a flexible genetic mechanism to rapidly changing the timing of ossification. Facial length is co-regulated with mastication, which is a product of muscle forces. Changes in bone length thus probably reflect a co-evolution of muscles and bones and suggest a pleiotropic role for Runx2 in mammalian evolution (Sears et al., 2007).

Furthermore, muscle strains are needed for fracture repairs. According to Hao et al. (2012), bony union was affected in rats whose quadriceps were treated with botulinum toxin-A (BXTA); saline was injected into the contralateral quadriceps. At different time points up to 8 weeks post-fracture, a gap was still visible on X-ray images of that side and no mature osseous calluses or woven bone were found by histology on the BXTA-treated side. Finally, biomechanical testing indicated that the femora of the BXTA-treated side exhibited inferior mechanical properties compared with the control side.

Beyond an obvious biomechanical importance of muscle strains for fracture repair, locally-released factors seem playing a role: covering bone fractures with muscle hastened healing and resulted in an increase in union strength. Thus, muscle-derived stromal cells exposed to an adjacent fracture differentiate into osteoblasts *in vitro*; the osteogenic potential of these cells exceeds that of adipose and skin-derived stromal cells and is equivalent to bone marrow stromal cells. The recruitment and differentiation of muscle-derived stromal cells in response to tumor necrosis factor-α (TNF-α) was shown to promote an accelerated healing of the fracture in an *in vivo* murine model (Glass et al., 2011). A local injection of recombinant human TNF-α on the first two days after fracture-inducing surgery accelerated healing of the fracture. By day 28, TNF-α treated animals showed significantly higher callus mineralization compared to controls. Importantly, TNF-α is one member of a large family of inflammatory cytokines that share common signal pathways, including activation of the transcription factor Nf-κ B. NF-κ B is a key regulator of inflammation in skeletal muscle (Peake et al., 2010), thus plays a critical role in muscle atrophy. As a skeletal catabolic agent, TNF-α stimulates osteoclastogenesis while simultaneously inhibiting osteoblast function (Nanes, 2003). Paradoxical role of TNF-α in fracture repair is interesting, since it probably points out at a necessity of resorption of bone edges and the regulated apoptosis in the callus for its ossification, osteon integrity, and thus an efficient fracture healing.

Both bone and muscle show major changes during aging and in the same direction, with sarcopenia and osteoporosis contributing to frailty (Matthews et al., 2011). Similarly to bone, muscle tissue deteriorates with the age. Sarcopenia term has been coined which is based on lower muscle quality, just as osteoporosis is typified by both decreased bone mass and structural integrity (Matthews et al., 2011). In addition, based on the mechanostat theory, the muscle-bone unit has been defined as a functional system whose components are under the common control of several hormones (Zofkova, 2008). Sex steroids modulate the function of the muscle-bone unit in adulthood and aging. Therefore, based on theoretical principles of allometry and the ample empirical data on co-regulation (provided above), the existence of genes determining characteristics of both traits is plausible (Karasik and Kiel, 2010).

### Genetic correlation between the muscles and bones and quantitative trait loci affecting both

Evidence for genetic correlations was described in the literature between geometric parameters of femoral bone neck and total body lean mass in men and women (Sun et al., 2006). It is not surprising, since lean mass was positively associated with section modulus and cross-sectional area in both sexes (*r* = 0.36–0.55, *p* < 0.05) (Moseley et al., 2011). Deng et al. (2007) performed a bivariate genome linkage analysis which produced two chromosomal regions, 5q35 and 10q24, with pleiotropic effects on these phenotypes. Results in the Framingham Osteoporosis Study demonstrated similar bivariate genetic correlations between leg lean mass and cross-sectional femoral geometry; bivariate linkage analysis identified significant quantitative trait loci (QTL) of leg lean mass shared with shaft CSA on chromosome 12p12–12p13 and with neck shaft angle, on 14q21–22 (Karasik et al., 2009). One cannot exclude that a third trait (such as fat mass) might be involved in pleiotropic relationships between bone and muscle, probably even as a mediator of their relationship.

There is an importance studying complex diseases and aging related processes through large animal models and translate animal research findings to future biomedical sciences. As of now, mostly small organisms are being used for this purpose, for example, zebra fish are being explored as a model for investigating the effect of aging on male reproduction (Kanuga et al., 2011). In livestock, we only find genetic evidence for skeletal muscle mutations which affect muscle growth and development but the effect on aging is not yet being explored. In sheep for example, the *callipyge (CLPG)* mutation causes post-natal muscle hypertrophy, which is localized in the pelvic limbs and loin (Cockett et al., 2005). Muscles from *CLPG*-expressing lambs enlarge to different degree and not all muscles are affected (Cockett et al., 2005). The *CLPG* trait in sheep exhibits a novel mode of inheritance termed “polar over-dominance” while the only animals that express the *CLPG* phenotypes are those heterozygotes who inherited the *CLPG* mutation from their sire (Georges et al., 2003). Enhanced skeletal muscle growth is also observed in animals with the *Carewell* (or *rib-eye muscling)* mutation, and a double-muscling phenotype has been documented for animals of the Texel sheep breed (Cockett et al., 2005). QTL were identified for traits related to bony carcass and meat quality in sheep while genome-wide significant QTLs were mapped for muscle (Karamichou et al., 2006) and bone (Campbell et al., 2003) densities. Since clinical studies are not easy to be implemented, large animals can be used as models to explore aging-related complex diseases and their effect on the musculoskeletal system. Future identification of the actual genes or mutations responsible for these bone and muscle related QTLs in livestock and especially their role in aging, will increase the understanding of vertebrate musculoskeletal biology.

### Pleotropic relationships between bone and muscle: is this a “true” pleiotropy or a mediation?

**Figure 2:**
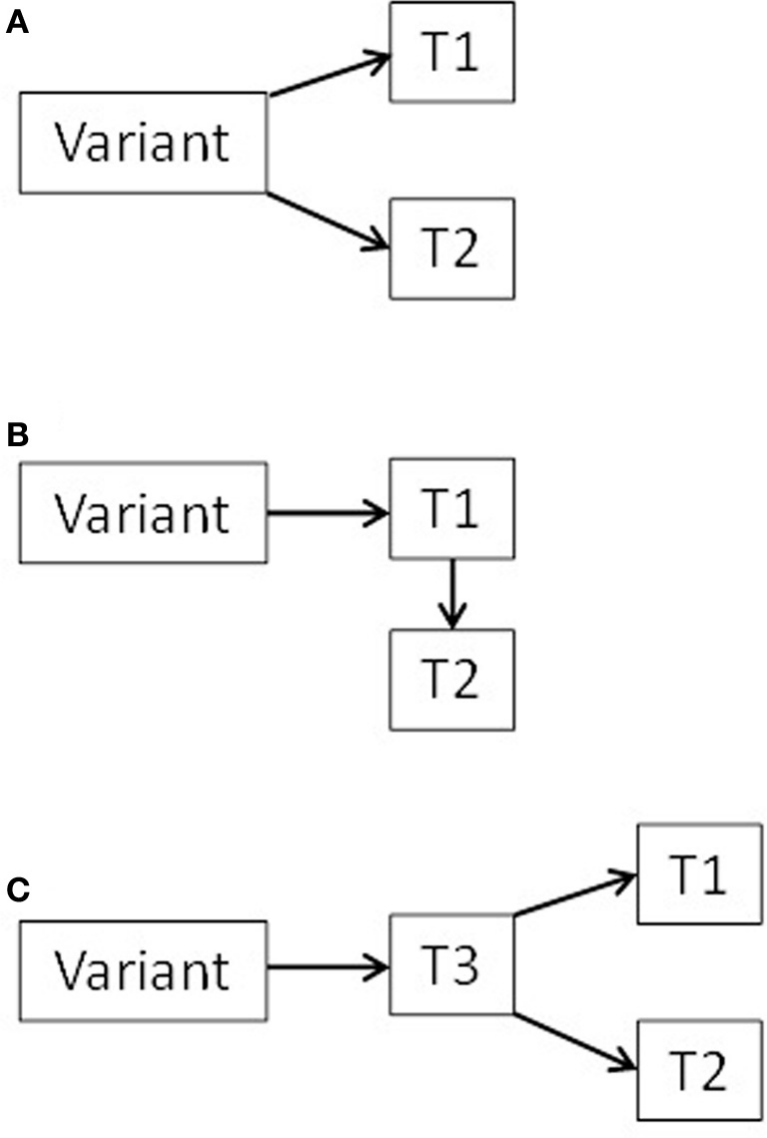
**Diagram of three possible scenarios (A, B, and C) for pleiotropic bone-muscle interactions (*T* = trait)**.

There are several potential mechanisms underlying associations between genetic variants and the parts of the musculoskeletal apparatus, including the mediation of environmental influences by genetic factors (Karasik, 2011). It was previously postulated that the spectrum of pleiotropic effects of a gene on a morphological trait may include direct or indirect effect with a possible continuum in-between (Kelly et al., 2006). We previously mentioned two possible scenarios of gene actions. The first one includes pleiotropic effects of gene polymorphisms on two traits, Trait1 and Trait2 (say, muscle and bone), and the second includes a conditional model (mediation), in which a gene is associated with one trait, and that trait in turn influences or affects another trait. (see scheme in Figure 2, scenarios **A** and **B**). Additional possible scenario can be suggested where a gene is affecting a third—unknown—trait (Trait3), which in turn affects directly both muscle and bone (confounding). In any case, products of the same gene(s) can be used as biomarkers for two traits simultaneously.

An example for cumbersome relationships when pleitropy is suspected, comes from a naturally occurring recessive mutation, named “mini muscle” (MM), that causes some 50% reduction in hind limb muscle mass of mice (Kelly et al., 2006). Observed reduction of both muscle mass and bone structure could represent direct pleiotropic effects of the MM allele if the gene is expressed in the stem cell populations early in development. Alternatively, the gene might act intrinsically only on the muscle cells and not the bone cells, and the MM bone phenotype could arise via weaker mechanical input from the reduced hind limb musculature (Wallace et al., 2012).

Pleiotropy in humans is ubiquitous (Sivakumaran et al., 2011); there are multiple examples in the literature, starting with a textbook example of phenylketonuria (PKU), caused by a deficiency of the enzyme phenylalanine hydroxylase, which is necessary to convert the essential amino acid phenylalanine to tyrosine. A defect in the single gene that codes for this enzyme therefore results in the multiple phenotypes associated with PKU, including mental retardation, eczema, and pigment defects that make affected individuals lighter skinned (Paul, 2000). In the most recent study, a single nucleotide polymorphism (SNP) rs11655470 in the region where the *CRHR1-MAPT* genes are located was related to infant head circumference (Taal et al., 2012). Variants in or near *CRHR1* have been previously associated with brain development and BMD (Rivadeneira et al., 2009). In a parallel paper, Ikram et al. (2012) showed that a correlated SNP in the same region (rs9303525; linkage disequilibrium, LD, *r*^2^ = 0.22 with rs11655470) is associated with adult intracranial volume. Since LD between the variants found to be low, it is possible that they represent separate, independent signals. After a series of statistical models with adjustments of one trait for the other, the investigators concurred that these signals reflect a third marker influencing both phenotypes, the head circumference and intracranial volume. In the above example of the MM allele, the authors similarly rise a possibility that there exists a circulating hormone or growth factor that regulates both muscle and bone development (Wallace et al., 2012). As occurs when the confounded scenario is a correct one, frequently there is a third SNP which seems to be associated with both traits, whose relationship with a combination of traits is stronger than with either trait. The biological causality underlying the potentially-pleiotropic associations is largely unknown, and cannot be resolved with statistical means; the definite answer should come from the “bench”—functional studies.

## Using genomic tools in order to detect genetic variants involved in pleiotropic mechanisms

Recently, in parallel with the upsurge of the whole-genomic genotyping and sequencing techniques, large-scale analyses and data mining methods have been developed to identify associations at a genomic level. These high-throughput techniques have enabled genome-wide association studies (GWAS), in which about 100,000—1,000,000 SNPs (representative of millions of SNPs in human genome) are tested. Linkage studies, GWAS and re-sequencing as well as new bioinformatic tools pointed out some interesting musculoskeletal genes, whose, in turn, contribute to identifying new pathways of possible pleiotropic value. We summarized the candidate genes with plausible biological pleiotropic effects on muscles and bones, as well as other tissues, in Table 2. For example, a possible candidate for pleotropic effect between bone and muscle is α-actinin 3 (*ACTN3*) gene which is highly expressed in fast skeletal muscle fibers (Yang et al., 2011). ACTN3 is one of the two isoforms of α actinin which are found in Z-discs of skeletal muscle (Chan et al., 2008; Yang et al., 2011). Genetic studies previously suggested that the absence of ACTN3 is detrimental to sprint and power performances in elite athletes and in the general population (Chan et al., 2008; MacArthur et al., 2008). Yang et al. (2011) recently showed that ACTN3 deficiency is associated with reduced bone mass in humans and mice.

**Table 2:** **Candidate genes and pathways with pleiotropic action on the musculoskeletal system [modified from (Karasik and Kiel, 2010) with permission]**.

**Gene (abbrev.)**	**Gene title (alias)**	**Action**	**Reference**
**SEX HORMONES**			
*AR*	Androgen receptor	Decreased AR activity results in a loss of bone mass	Bhasin and Buckwalter, 2001
		*CAGn* repeats associated with fat-free mass in men	Walsh et al., 2005
*ESR1*	Estrogen receptor 1	In a meta-analysis, *XbaI* polymorphism associated with BMD and fracture risk in women	Ioannidis et al., 2002
		*Esr1* knock-out mice unable to respond to physical exercise with a periosteal bone expansion compared to wildtype mice	Lee et al., 2003
		*PvuII* polymorphism may modulate the effect of exercise on BMD	Suuriniemi et al., 2004
		Note: no relationship between *TA*-repeat polymorphism and muscle mass and strength in young adult women	Grundberg et al., 2005
*COMT*	Catechol-O-methyltransferase	Val158Met polymorphism was associated with peak BMD in young men and an interaction of *COMT* with physical activity on BMD was found in the same young men	Lorentzon et al., 2004, 2007
		Girls with *COMT*^*LL*^ compared to *COMT*^*HH*^ genotype had more lean mass as measured by DXA, and an increased muscle area in the tibia as measured with pQCT	Eriksson et al., 2005
		mCSA differed by Val158Met genotypes (significantly larger in LL than HL individuals) in *older Finnish women*	Ronkainen et al., 2008
**GROWTH HORMONE/INSULIN-LIKE GROWTH FACTORS**			
*IGF1*	Insulin-like growth factor I	CA-repeat promoter polymorphism has effects on femoral bone geometric parameters	Rivadeneira et al., 2004
		CA-repeat polymorphism was associated with increased bone strength and muscle volume and strength	Kostek et al., 2005
		Alternative splicing was involved in the mechanotransduction of bone cells	Goldspink and Yang, 2004; Tang et al., 2004
**TRANSFORMING GROWTH FACTOR-β SUPERFAMILY**			
TGFB1^*^	Transforming growth factor-β 1	Association of 29C > T polymorphism in the transforming growth factor-β 1 gene with lean body mass in community-dwelling Japanese population	Fuku et al., 2012
		SNPs in *TGFB1* found to be associated with peak bone mass in young healthy Caucasian females	Tzakas et al., 2005
MSTN^*^	Myostatin	Myostatin-null mice had significantly greater cortical bone mineral content and larger entheses than normal mice	Hamrick et al., 2003; Hamrick, 2003
		mRNA levels were reduced in response to heavy-resistance strength training in older adults	Roth et al., 2003
		SNPs (rs2293284 and rs7570532) were associated with hip peak BMD variation in Chinese women	Zhang et al., 2008
**VITAMIN D SIGNALING**			
*VDR*	Vitamin D receptor	In a meta-analysis, *Cdx-2* polymorphism was associated with risk for vertebral fractures in women	Uitterlinden et al., 2006
		*BsmI* polymorphism was associated with decreased vertebral area and femoral narrow neck width	Fang et al., 2007
		*BsmI* polymorphism was associated with muscle strength	Windelinckx et al., 2007; Grundberg et al., 2004
		*FokI* polymorphism was associated with fat-free mass and sarcopenia in older men	Roth et al., 2004
		Interactions between leisure physical activity and VDR *BsmI* genotype on the lumbar spine BMD in active post-menopausal women	Blanchet et al., 2002
		Association between *VDR* polymorphisms and falls, balance and muscle strength	Barr et al., 2009
**INFLAMMATORY CYTOKINES**			
*IL6*	Interleukin 6	*–174 GC* polymorphism was associated with increased risk of wrist fracture in post-menopausal women and with hip BMD in post-menopausal women	Nordstrom et al., 2004; Ferrari et al., 2004
		*–174 GC* was associated with fat-free mass in men but not women	Roth et al., 2003
		Exercise increases IL-6 receptor production in human skeletal muscle	Keller et al., 2005
*IL15*	Interleukin 15	Transgenic mice (overexpressing IL-15 in skeletal muscle, with elevated circulating levels) show increased bone mass	Quinn et al., 2009
*TNF*	Tumor necrosis factor (a.k.a. TNF-α)	*TNF* promoter polymorphisms associated with muscle phenotypes in humans *TNF* negatively regulates bone formation in rats	Liu et al., 2008 Zhou et al., 2006
**OTHER PATHWAYS**			
*BMP2*	Bone morphogenetic protein-2	Young males with the *rs15705* C/C genotype were associated with an increased gain in skeletal muscle volume (*P* = 0.0060) following resistance training	Devaney et al., 2009
		*BMP2* was linked and associated with BMD at different skeletal sites	Xiong et al., 2006; Styrkarsdottir et al., 2003
PPARG^*^	Peroxisome proliferator-activated receptor γ	Polymorphisms in the PPARγ were associated with aBMD in both mice and humans Mutations in PPARγ result in increased fatty acid flux to the skeletal muscle	Ackert-Bicknell et al., 2008 Savage et al., 2003
*MEF2C*	Myocyte enhancer factor 2C	Responsible for controlling bone development, by activating chondrocyte hypertrophy.	Arnold et al., 2007
NR3C1^*^	Nuclear receptor subfamily 3 group C member 1 (a.k.a. Glucocorticoid receptor)	Contributed both to bone and lean mass in older persons, muscle strength in younger males *ER22/23EK* polymorphism was associated with lower trochanteric BMD in elderly women	van Rossum et al., 2003, 2004 van Schoor et al., 2007
*PTN*	Pleiotrophin	Over-expression affects mouse long bone development, fracture healing and bone repair	Li et al., 2005
		Potential mediator of mechanotransduction signaling in regulating periosteal bone formation and resorption in mouse	Xing et al., 2005
		Expression levels lowered in response to spaceflight	Nikawa et al., 2004
*NOTCH1*	Notch homolog 1, translocation-associated	NOTCH1 inhibits bone resorption, both directly on osteoclast precursors and indirectly via osteoblast lineage cells	Bai et al., 2008
		Significantly lower expression found in muscle biopsies from older men compared to muscle from younger men	Carey et al., 2007
*NOTCH2*	Notch homolog 2, neurogenic locus notch homolog protein 2	Mutations in NOTCH2 cause Hajdu-Cheney syndrome, a disorder of severe and progressive bone loss	Simpson et al., 2011
*RETN*	Resistin	Serum levels showed a significant negative correlation with lumbar spine BMD in middle-aged men	Oh et al., 2005
		Polymorphisms associated with muscle and bone phenotypes in men and women	Pistilli et al., 2007
*SOX17*	Transcription factor SRY (sex determining region Y)-box 17	Involved in endochondral bone growth Downregulated in older men (as a part of the “sarcopenia signature”)	Agoston et al., 2007 Giresi et al., 2005
*SOX6*	(sex determining region Y)-box 6	Associated with BMD in GWAS studies Expressed in a wide variety of tissues, most abundantly in skeletal muscle	Liu et al., 2009; Rivadeneira et al., 2009 Smits et al., 2001
*NFKB1*	Nuclear factor of kappa B (a.k.a. *NF-kB*)	NF-kB proteins implicated in muscle wasting (short-term hindlimb unloading in rodents) Activation of NF-kB can induce muscle atrophy in transgenic mice	Kandarian, 2008 Cai et al., 2004
*LMNA*	Lamins A/C	Mutations cause primary laminopathies, including skeletal muscular dystrophies	Jacob and Garg, 2006
		Lamin A/C knockdown had a negative impact on osteoblastogenesis and bone formation *in vitro*	Akter et al., 2009
*FOXO1*	Forkhead box protein O1	Skeletal muscle transgenic mice (FKHR) have less skeletal muscle mass, down-regulated type I fiber genes and impaired glycemic control	Kamei et al., 2004
		Positive regulator of bone formation, required for osteoblast proliferation	Rached et al., 2010
*VCP*	Valosin-containing protein	Mutations cause inclusion body myopathy with early-onset Paget's disease and frontotemporal dementia (IBMPFD) syndrome	Watts et al., 2004
		Transgenic mice expressing mutant forms VCP/p97 recapitulate the full spectrum of IBMPFD syndrome including degeneration in muscle, brain and bone.	Custer et al., 2010
*ACTN3*	α-Actinin-3	Muscle deficiency is detrimental to sprint and power performance in humans Deficiency is associated with reduced bone mass in human and mouse	Chan et al., 2008; MacArthur et al., 2008 Yang et al., 2011

* According to GeneCards (http://www.genecards.org)—Last accessed on April 29*th* 2012.

As follows from the Table, along with the biological candidates, some genetic studies produce “serendipitous” pleiotropic findings. Similarly, genetic association studies for rare diseases have their merit by uncovering some facets of the complex musculoskeletal biology. Often methodologically different, both belong to the domain of human genetic study. Here are examples of recent discoveries of genes with a potentially-pleiotropic role in the musculoskeleton.

In a large GWAS performed by the GEFOS consortium, a locus at 5q14—near myocyte enhancer factor 2C (*MEF2C*)—was found to be significantly associated with BMD related traits (Rivadeneira et al., 2009). Besides being involved in myogenesis (Cesana et al., 2011) like the other myogenic basic helix-loop-helix proteins, members of MEF2 family of regulatory proteins are participating in bone-relevant pathways including endochondral ossification (Kramer et al., 2012). Notably, by virtue of being widely pleiotropic, *MEF2C* was associated with different traits, such as platelet count, retinal vascular caliber, tonometry, and adult height. Future research should be performed to decipher a “real” musculoskeletal pleiotropy of this molecule, as opposed to a mediation effect via some primary basic mechanism active early in development.

Another segue, now into the pathobiology of tendons, was provided by a recent GWAS of Dupuytren contractures (Dolmans et al., 2011). Dupuytren's disease is a fibromatosis of the flexors, whose prevalence increases with age and leads to flexion contractures affecting fingers. Six of the 9 loci identified by the study included genes known to be involved in the Wnt-signaling pathway, including *WNT4, WNT2, WNT7B, SFRP4, SULF1*, and *RSPO2.* The latter gene encodes R-spondin, a member of the family interacting with frizzled receptors and LRP5/6 to induce β-catenin signaling; *Rspo2* expression is required for Wnt11-mediated osteoblast maturation. Wnt-signaling pathway is among the leading regulators of bone mass, as is shown time and again (Rivadeneira et al., 2009; Zhang et al., 2010; Estrada et al., 2012). In muscle, a conversion of myogenic into fibrogenic lineages could be abrogated experimentally by treating mice with Wnt inhibitors (Mann et al., 2011). Similarly, injection of the Wnt antagonist DKK1 into the skeletal muscles of *mdx* mice (see below) significantly reduced fibrosis. Indeed, there was reduced fibrosis and enhanced muscle regeneration in aged muscle injected with DKK1, whereas no change was observed in young similarly treated muscle (Brack et al., 2007). A mechanism of Wnt-induced cell-fate changes from myogenic to non-myogenic cells in resting satellite cells awaits further validation (Mann et al., 2011). Notably, a role of another important Wnt antagonist, SOST in skeletal muscle has not been explored yet.

Other evidence for the importance of muscle-to-bone cross-talk in bone health and disease comes from the rare diseases of muscles. Thus, DMD is an X-linked disease where the gene encoding the protein dystrophin is affected by various mutations. Fractures are a significant problem in patients with DMD (young boys). Two recent studies investigated bones affected by the deficiency of dystrophin, which is mutated in DMD. Both used dystrophin-deficient *mdx* mice, a model of human DMD. Thus, one study (Novotny et al., 2011) showed that tibiae of *mdx* mice had up to 50% lower strength and stiffness compared to wild-type mice; they had reductions in cortical cross-sectional moment of inertia, cross-sectional area, and in trabecular bone volume. Importantly, this compromised bone strength was already obvious in very young mice (Novotny et al., 2011), which corresponds to poor bone health in DMD boys. The second group (Nakagaki et al., 2011) investigated the changes that occur in the femur of young *mdx* mice, at 21 days of age. They also demonstrated a lower strength, stiffness and energy absorption capacity in *mdx* femora, which were shorter, had a smaller cortical area and thickness, and manifested changes in the ECM and collagen organization. Interestingly, at 3 weeks of age the muscle damage in mice was still not significant, thus it is a lack of DMD expression, even in the absence of significant muscle fiber degeneration, which seems to be affecting bones (Nakagaki et al., 2011). The underlying molecular mechanisms for the effects on bone, whether direct or indirect, have not yet been elucidated in detail, which makes this exploration tantalizing. The above—semi-serendipitous—findings of pleiotropy, call out for a systematic study dedicated to identifying genetic variants underlying muscle and other related traits, for shared genetic mechanisms underlying parts of the musculoskeleton. In the next sub-section we discuss a framework that may be useful in analyses of the “-omics” data.

### Post-GWAS and gene expression studies

Our knowledge of the genetic architecture of common musculoskeletal diseases remains rudimentary, in part due to the complex relationship between the phenotype and genotype (and the environmental effects). Only recently has it been possible to address the need for high throughput gene expression studies (RNA-seq, transcriptome) focusing on multiple relevant tissues, such as differential expression of skeletal and muscular genes with aging. Transcriptional profiles of many human tissues, including muscle (Welle et al., 2004; Zahn et al., 2006; Wallace et al., 2012), skin (Lener et al., 2006), tendons (Jones et al., 2006), and cartilage (Swingler et al., 2009); have been generated; however, for human bones, there are fewer available resources (Grundberg et al., 2008; Reppe et al., 2010).

Mantila Roosa et al. (2011) study used the rat forelimb loading model to evaluate the extent of alternative splicing in bone under mechanical loading. Animals were subjected to loading sessions every day, and ulnae were sampled at 11 time points, from 4 h to 32 days since loading started. They identified multiple alternatively spliced genes encoding cytokines, ion channels, solute carriers, and notably, muscle-related genes. Previously, Paic et al. (2009) isolated osteocyte and osteoblast cell populations for microarray analysis. Multiple muscle-related genes were downregulated in osteoblasts with respect to osteocytes, including many of the same genes downregulated by loading in the study of Mantila Roosa et al. (2011). Although the involvement of muscle-related genes and proteins in bone biology is not well understood, it is clear that they are highly regulated in bone cells. One can speculate that finding of the muscle-related genes being downregulated both during bone formation and in osteoblasts compared to osteocytes is linked to mechanosensitivity, which is characteristic of osteocytes.

Transcription factors that play a role in ossification during development are expected to participate in post-natal fracture repair since the endochondral bone formation that occurs in embryos is recapitulated during fracture repair (Reumann et al., 2011). The knowledge of their interplay during the bone formation, as well as functioning as parts of muscle-bone unit, should provide the strategic basis for interventions aimed at improving tissue repair after fracture, by mechanical stimuli or some humoral factors, or by a combination of both.

Several studies have used gene profiling to identify gene clusters and individual genes in muscle that change with age (Roth et al., 2002; Giresi et al., 2005; Dennis et al., 2008, 2009; Thalacker-Mercer et al., 2010). Furthermore, expression profiling was used to identify genes expressed in adult rat and human tendon tissue (Jelinsky et al., 2010). Using this technique, approximately 1,600 transcripts appeared to be selectively expressed in rat tendon tissue and approximately 300 transcripts appeared to be selectively expressed in human tendon tissue, with ~20 genes overlapping between both human and rat tendon tissue. Of these common tendon-selective genes, thrombospondin-4 and tenomodulin were found to have the highest tendon-selective expression compared to other tissues examined. Interestingly, expression of these tendon-selective genes, which are present in primary tendon fibroblasts, is lost when these cells are placed in two-dimensional culture systems (possibly suggesting that mechanoception is a factor for expression of these genes). Identification of tendon-selective genes provides potential molecular tools to facilitate a better understanding of tendon development and repair.

Drummond et al. (2011) profiled microRNA (miRNA) expression patterns in aging human skeletal muscle followed by in-depth functional and network analysis. In a classical fashion of such experiments, muscle biopsy samples from 19 younger men (age 31 ± 2) were compared with the older (73 ± 3 yrs old; *n* =17). Eighteen miRNAs were differentially expressed, including Let-7 miRNA family members, Let-7b and Let-7e (Drummond et al., 2011). A higher expression of Let-7 family members that may down-regulate genes related to cellular proliferation fits well into our understanding that older human muscle is characterized by reduced muscle cell renewal and regeneration.

Expression QTLs (eQTLs) can be used as a tool assessing genetic regulation of the expression levels of mRNA in desired tissues. These eQTLs can be mapped to a variant in a local gene (*cis* eQTLs) or to variant on a different chromosome (*trans* QTLs). In porcine, identification of eQTLs of genes expressed in longissimus dorsi found to be associated with meat quality traits (Ponsuksili et al., 2010). As an example for the adipose tissue, Small et al. (2011) and the MuTHER consortium demonstrated that the Type 2 diabetes and HDL-cholesterol associated *cis*-acting eQTL of the maternally-expressed transcription factor *KLF14* acts as a master *trans*-regulator of adipose gene expression. Expression levels of genes regulated by this *trans*-eQTL are highly-correlated with concurrently-measured metabolic traits, and a subset of the *trans*-genes harbor variants directly associated with metabolic phenotypes. The authors suggested that by leveraging “-omics” data from multiple sources they are able to discover new biological and functional insights. A note of caution should be sounded while interpreting these experiments, since the cell culture and animal models may not reflect the complex dynamic interactions between genes and the environment that form the basis of human phenotype (Franceschi et al., 2007).

## Conclusion

The pathobiology of many of the musculoskeletal diseases remains obscure, as do factors affecting disease severity (Laing, 2012). Thus, for example, the establishment of new therapies, such as regenerative medicines, for injured tendons has been delayed by a limited understanding of tendon biology (Ito et al., 2010). Several markers have been shown to correlate with the burden of musculoskeletal diseases; additionally new techniques must be developed to identify and quantify the biomarkers in order to support both genetic diagnostics and a genetic study, which is a powerful tool in biological discovery. We need to focus on genetic aspects of the cross-talk between muscle and its “neighboring” tissues, to find previously unknown pathways of inter-compartmental communication. Multivariate methods for large-scale data analysis and mining of results were proposed and validated (Shriner, 2012). Ideally, a multivariate GWAS, identifying genetic variants underlying both bone and muscle, should be replicated in large human cohorts. Similarly, tantalizing findings of pleiotropy between the genes regulating muscle and energy metabolism, insulin resistance, fat depots, and reproductive aging, should be explored further.

Several studies have been proposed in this Perspective that could address the areas of immediate interest, and the key questions can be summarized as follows. Focus should be on a possibility that growth factors (inflammatory cytokines, myokines, etc.) expressed in muscle could affect signaling in bone cells. New methods are needed to accurately measure the *in vivo* mechanical responses and in particular, how aging may affect the ability of mechanical loading to stimulate muscles by activating satellite cells (Thomis and Aerssens, 2012) and further trigger anabolic responses in bones (Wu et al., 2011). Therapeutic applications of the biological knowledge of muscle-bone-cartilage interface can further be extended to the fractures: risk prevention by soft-tissue cushion, fracture repair by adjacent muscles, or improving tendon-bone attachment in orthopedics. This knowledge would help institute treatment interventions aimed at improving bone tissue repair and successful regeneration of healthy muscle, thus reducing adverse outcomes in vulnerable populations, such as aged people and others (e.g., professional athletes). Combined strategies will be crucial to ameliorate muscle loss with the ensuing inflammation, fatty infiltration, and fibrosis, including systemic delivery of anti-inflammatory or anti-aging agents and gene-corrected cells, adapted for the local milieu (Mann et al., 2011). More fundamental study is desired to learn how aging contributes to musculoskeletal diseases. Answers to these questions will vastly improve our understanding of normal function of the musculoskeleton as a whole. Finally, identifying mechanisms by which etiologies of several diseases are interconnected, will provide potential targets for systemic therapeutic interventions. From a general gerontological perspective, a rapidly aging system such as the musculoskeletal provides challenges for studying its normal decline and trying to prevent it but also forthcoming rewards if our efforts succeed.

### Conflict of interest statement

The authors declare that the research was conducted in the absence of any commercial or financial relationships that could be construed as a potential conflict of interest.
